# Impact of Cardiopulmonary Resuscitation on Emergency Medical Staff—Romanian Perspective (IRESUS-EMS)

**DOI:** 10.3390/jcm11195707

**Published:** 2022-09-27

**Authors:** Paul-Lucian Nedelea, Mihaela Corlade-Andrei, Cristina Kantor, Ovidiu Tudor Popa, Emilian Manolescu, Diana Cimpoeșu

**Affiliations:** 1 Emergency “St. Spiridon” Hospital”, 700111 Iasi, Romania; 2 Faculty of Medicine, University of Medicine and Pharmacy “Grigore T. Popa”, 700115 Iasi, Romania

**Keywords:** cardiopulmonary resuscitation, ethics, emergency department staff, unnecessary CPR, quality of life

## Abstract

Background: Unnecessary resuscitation is defined as putting in a disproportionate amount of effort compared to the patients’ prognosis and chance of survival. The primary objective of this study was to determine the number of resuscitations perceived as unnecessary by emergency medical personnel and to correlate it with the characteristics of resuscitation team members, patient particularities and organizational factors related to the professional environment. Methods: This was a prospective cross-sectional study carried out in the emergency department of a university hospital, exploring the perception of the uselessness of cardiopulmonary resuscitation (CPR) through the completion of a questionnaire. Results: In total, 70.37% of respondents are often involved in CPR attempts in which the efforts made are disproportionate compared to the patients’ expected prognosis, in terms of survival or quality of life. The presence of a non-shockable rhythm increased, by two times, the chances of medical staff finding it unnecessary to initiate CPR. Conclusions: The current study was the first in Romania to investigate the perception of unnecessary CPR, based on the recollection of the last resuscitation performed by the emergency medical staff. The objective criteria related to the patient were the most important predictors for assessing the adequacy of the decision to initiate CPR.

## 1. Introduction

The World Association of Physicians defines “useless” treatment as treatment that “offers no reasonable hope of recovery or improvement” or from which the “patient” will never benefit. Resuscitation is considered unnecessary when the chances of a quality survival are minimal [1].

The first premise from which to start considering a certain treatment as useless is the presence or absence of a medical indication. The practice of current resuscitation is motivated by the assumption that in the absence of a previous desire, any patient wants to be resuscitated, at any cost and regardless of the chances of success, regardless of functional status after resuscitation or comorbidities [2,3].

Initiating unnecessary treatment can create false hope to both the family and the patient, hope that in turn is liable to undermine the patients’ ability to make a rational judgment and to threaten their autonomy [1,4]. However, decision makers have the duty to consult with the patients or their representatives, if the former are unable to make such decision, in accordance with “a clear and accessible policy” [5,6,7].

Although some studies suggest that, in recent years, the neurological evolution after CPR has improved, this has not been proven in cardiac arrest (CA) patients with an initial non-shockable rhythm, where the survival rate is 3 to 15 times lower than in arrests where the initial rhythm is shockable, which accounts for approximately 23% of CAs reported in the European, American and Canadian registries and only 6–8% in Japan [8,9,10,11].

There may also be situations in which clinicians may perceive cardiopulmonary resuscitation (CPR) as inappropriate. An anonymous survey of randomly selected emergency physicians in the United States shows that 55% of them performed resuscitation more than 10 times in the past 3 years, despite their professional opinion of futility. In total, 98% of them tried resuscitation in the case of terminally ill patients, a situation in which they personally would not have wanted resuscitation [12]. Of the medical staff working in the prehospital, 49% stated that they ignored the patient’s previous wish not to be resuscitated because they could not fail to perform the medical interventions for which they were prepared [13].

The clinician’s perception of inappropriate CPR is important because it has been associated with impaired morale [14,15] and psycho-emotional exhaustion (the burnout effect) [16], which has been linked to suboptimal levels of patient care. Thus, repetitive CPR attempts that are perceived as inappropriate and which have a negative outcome may adversely affect the quality of the subsequent resuscitation attempts [17,18]. The presence of a correlation between the clinician’s perception of inadequate CPR and the outcome of the patient’s resuscitation provides a basis for arguing the usefulness of the clinician’s perception and its value in resuscitation practice.

The primary objective of this study was to determine the number of resuscitations performed in the hospital or in the emergency department perceived as unnecessary by the medical staff and correlated with the characteristics of the resuscitation team members, the patients’ particularities and the organizational factors related to the professional environment and return of spontaneous circulation (ROSC), as well as the survival at discharge. Unnecessary resuscitation is defined as putting in a disproportionate amount of effort compared to the patients’ prognosis and their chances of survival.

The secondary objectives were to assess the level of stress and determine the intention of the staff who participated in the study to leave the current workplace of the emergency medical services.

## 2. Materials and Methods

### 2.1. General Methodology

The IRESUS-EMS study was a prospective cross-sectional study, conducted in the Emergency Department (ED) of the “St. Spiridon” County Emergency Clinical Hospital of Iași in 2015, that explored the perception of the uselessness of cardiopulmonary resuscitation (CPR). The data analyzed in this study are part of the data collected and reported by Romania as a participant in the REAPPROPRIATE study (an international, prospective, multicenter cross-sectional study that took place in 288 centers from 24 countries). The European study coordinator allowed the use of anonymized national data by the research teams in their own studies and publications, after the publication of the multicenter study.

### 2.2. Study Population

The studied population comprised the following staff categories: doctors and nurses employed by ED-SMURD at “St. Spiridon” Emergency County Clinical Hospital, directly involved at the time of completing the questionnaire in the treatment of cardiorespiratory arrest in the ED or in the prehospital.

### 2.3. Data Collection

The study was based on a questionnaire validated by the APPROPRICUS study [19] and adapted to the field of emergency medicine by special sociological techniques. The final English-language questionnaire was translated into the language of each participating country using an adapted Brislin method to achieve cultural and functional equivalence [20]. Data were collected from an online questionnaire opened by the European REAPPROPRIATE study between March and November 2015. The Romanian data were gathered from August 2015, after receiving approval from the Ethics Committee of County Emergency “St. Spiridon” Hospital (protocol code 40290/19 August 2015).

### 2.4. The Questionnaire

A 4-point rating scale, from “Strongly Agree” to “Strongly Disagree”, similar to the Likert scale, was used to complete the questionnaire. The ED staff, doctors and nurses, were asked about their demographics, professional experience and the ED work environment. They were also asked to recall the latest cardiac arrest they faced (without need of the patients’ medical record in order to answer these questions) and to answer if they fully “agreed with the initiation of the resuscitation” (i.e., their own perception of the appropriateness of CPR), if they were “uncertain that resuscitation should have started” (uncertainty about the usefulness of CPR) or if they were “certain that resuscitation should not have started” (inappropriateness of CPR). Subsequent questions dealt with the details of the last recalled resuscitation circumstances.

### 2.5. Statistical Analysis

Data were analyzed using the SPSS V.24 software (IBM Statistical Package for the Social Sciences, Chicago, Illinois). The variables used in the multivariate analysis model are: variable characteristics of medical staff (demographic, educational, working conditions) and variable characteristics of the job (hospital/ED environment, presence of psychological support, resuscitation team structure or ethical environment).

The statistical study addressed two aspects: descriptive statistics and analytical statistics. Descriptive statistics show the synthesis of observational data. Statistically significant differences were considered for a 95% confidence interval, based on the results of the parametric or non-parametric tests applied. The significance level (*p*-value) representing the maximum probability of error was considered to be 0.05 (5%), with a probability (confidence interval) of 95%, indicating that the decision is fair.

To assess the perception of the uselessness of CPR, we used logistic regression, which provides a useful tool for modeling the dependence of perception on a series of explanatory variables called “predictors”, which can be categorical, ordinal or continuous. The analysis took into account the collinearity of the independent variables, which involved the verification of the intercorrelations between them. Thus, the variables selected and introduced as predictive factors did not show interdependencies (r < 0.2, *p* > 0.05, 95% CI).

The “enter” method, in which all of the predictors were included in one step, was applied, and the results of the Hosmer–Lemeshow test show that the model is appropriate. The value of R2 Nagelkerke indicates whether the model is useful in making predictions; although the explanatory contribution of two variables in the prediction is not statistically significant, the magnitude of the effect may be significant.

## 3. Results

### 3.1. Demographic Characteristics

Out of the 58 questionnaires distributed, 54 were completed by 27 physicians and 27 nurses who represented the basis of the analysis in the IRESUS-EMS study. The most recent CPR trial was reported by all 54 study participants. The study participants were very interested (87.04%) in this study; 55.6% were extremely interested while 31.5% were very interested. No significant differences were identified between nurses and physicians.

The ages in the study group ranged from 22 to 53, with a mean age of 34.9 years, and an SD of 7.2. The nurses who participated in the study had a significantly higher average age compared to the age of the doctors (*p* = 0.0107). Most of the sample comprised women (68.52%), with a significantly higher share (*p* = 0.0308) among nurses (81.48%) compared to doctors (55.56%). Of the respondents, 66.67% stated that they have a religious belief, which has an influence on current professional practice for 44.48% of respondents. A total of 51.8% of the study group were represented by emergency medicine residents.

### 3.2. Educational Characteristics

These characteristics were drawn from the number of hours of basic life support (BLS) or advanced life support (ALS) courses which they attended in the last 12 months, as well as the activity of BLS/ALS trainers and the evaluation of their training activity in cardiopulmonary resuscitation. The BLS course varied from 0 to 48 h, and in the case of doctors, a significantly higher value (*p* = 0.00001) for the number of course hours (11.5 ± 17.7 h) was observed. The number of hours of ALS proved to be similar to that of the BLS course. Out of the total number of respondents, only 14.81% stated that they were BLS or ALS course trainers, including some of the teachers of the Department of Emergency Medicine who carry out activities within the “Gr.T.Popa” University of Medicine and Pharmacy of Iași, but who actually practice emergency medicine in the hospital. With respect to CPR training activity, 85% of the participants stated that they benefited from specific training in cardiopulmonary resuscitation according to the current guidelines. Regarding the rapid recognition of those patients for whom resuscitation is inappropriate, 96.3% of the doctors agreed with it, compared to 81.4% of nurses. A lower percentage of doctors (85.19%) than nurses (96.3%) stated that they received training on collaborating within a multidisciplinary resuscitation team.

### 3.3. Working Conditions

The weekly clinical activity, the experience in the current service and the total seniority in emergency services were evaluated. The doctors had a significantly higher number (Z = 7.401, *p* = 0.008) of hours per week in clinical activity, with an average of 49.56 ± 10.7 h, compared to nurses (42.07 ± 5.24 h). The nurses participating in the study showed a significantly higher seniority at work (Z = 1.961, *p* = 0.047). The same proportion is maintained in the case of total seniority at work in the emergency service: the total number of years in the current job and in other similar jobs is significantly higher (Z = 2.85, *p* = 0.0043) in the case of nurses (10.4 years) compared to doctors (5.7 years). The average length of service was 8.1 years. In this analysis, the highest proportion was represented by respondents with 5–20 years of service (53.6%). On average, there are no significant differences (*p* = 0.4686) between the number of shifts worked by doctors (5.8 night shifts) compared to those worked by nurses (5.5 night shifts). In the case of nurses, there is a high frequency of those with seven night shifts per month, while in the case of doctors, a maximum frequency is recorded at five night shifts per month.

### 3.4. Relationship with Patients and the General Public

Generally, doctors experienced no communication problems relating to the patient’s culture, but there were some difficulties with nurses; 40.7% experienced such a problem, compared to 22.2% of doctors. The statement “I am appreciated for my work” was “agreed” or “totally agreed” with by a significantly higher number (*p* = 0.02912) of nurses (74.07%) than doctors (40.74%).

### 3.5. Workplace Tensions

Working in the resuscitation team is very hard work (*p* = 0.0238), which is felt especially by doctors. Doctors also stated, with a percentage of 51.85%, that time is not correlated with the required activities (*p* = 0.0022). The involvement of the staff of the resuscitation team was an aspect noted by all participants in the study. It was found that all the participants are willing to help when needed (*p* = 0.0261). Additionally, the percentage of nurses who considered the work they perform to be repetitive (44.44%), compared to doctors (22.22%) (*p* = 0.0181), is remarkable. In this context, the people involved in the study did not think about leaving their current profession (94.45%), but there were a small percentage of doctors (11.1%) who thought about it. These results are shown in Table 1.

### 3.6. Team Performance during Resuscitation

Recognizing one’s own value in the resuscitation team was a feature that was not felt by 85.18% of respondents (*p* = 0.0118).

It is also worth noting that the team members (88.9%) believed that they could not express their opinions during the resuscitation related to how appropriate that resuscitation attempt was. Additionally, the recognition of the efforts of the resuscitation team members by the team leader, regardless of the result, was identified in only 35.19% of cases.

Both doctors and nurses often asked themselves whether a resuscitation attempt (57.40%) was adequate, this being more frequent in doctors (59.26%). The presence of a family member during an attempt to resuscitate a person only occurred in 81.48% of cases (*p* = 0.012). Regarding the debriefing performed at the end of a resuscitation attempt, we identified this in 62.97% of cases. These results are presented in Table 2.

### 3.7. Perception of the Last Remembered Attempt of CPR

#### 3.7.1. Factors That Influence the Perception of the Inopportunity of Resuscitation

Of the factors associated with the perception of the inopportunity of the resuscitation, the only one with statistical significance was poor quality of life before the onset of CA, but in the sense that this factor was the least important in perceiving CPR as unnecessary (*p* = 0.0002).

In terms of the other studied factors—the general quality of the CPR initiated by witnesses (75.93%), the initial non-shockable rhythm (64.81%), the presence of a DNAR order (85.18%) and comorbidities such as the neoplasm (64.82%)—although they were considered important by a large proportion of respondents, we did not identify statistical significance for the influence on the perception of the uselessness of CPR. These results are shown in Table 3.

#### 3.7.2. Factors That Influence the Perception of the Uselessness of a CPR Attempt

Of the factors associated with the perception of the adequacy of CPR, the following were found to be extremely important: the presence of an initial shocking rhythm (85.18%) (*p* = 0.0122), the victim being a possible organ donor (88.89%) (*p* = 0.0154), the victim being a pregnant woman (96.29%) (*p* = 0.0194) or the victim being a child (98.15%) (*p* = 0.0161). These findings are highlighted in Table 4.

#### 3.7.3. The Last Cardiopulmonary Resuscitation in Which the Respondents Were Involved

Depending on the perception of resuscitation, it was found that 79.63% of the respondents considered it necessary to start resuscitation, 12.96% of them felt that it should not have been initiated and 7.41% of them were uncertain about the need to start resuscitation. A greater proportion of doctors than nurses felt that the resuscitation should not have been initiated (22.2% vs. 3.7%) (*p* = 0.0103).

**Figure 1:**
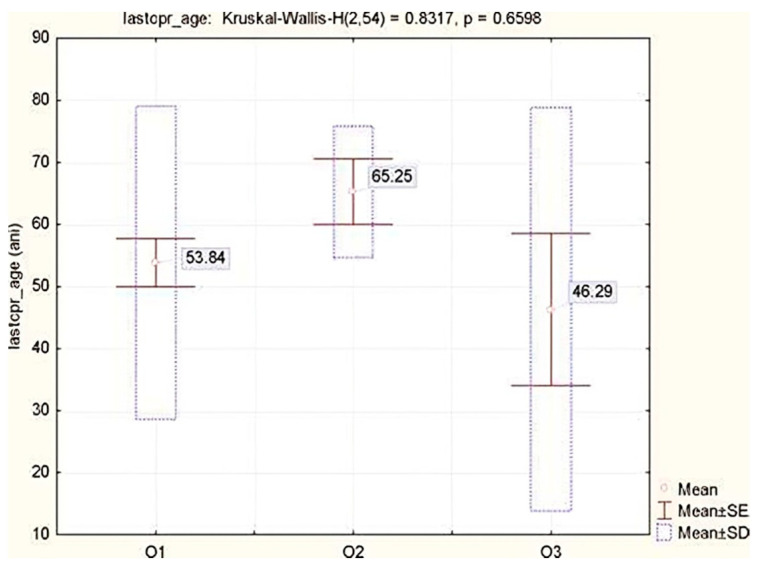
Average patient age according to the perception of the utility of CPR.

The ED was the location in which the last CPR attempt was held in 57.41% of cases. The share of the doctors who remembered their last CPR attempt as being in the hospital was 51.85%, significantly higher (χ^2^ = 5.893, *p* = 0.0167) than the share of nurses (33.3%). The ages of the patients for whom the last CPR was recalled ranged from 1 to 87, with a mean age of 53.7 years, and an SD of 25.4. The value of the 25% quartile indicates that 25% of patients were younger than 45 years. In total, 66.7% of patients were men. No significant difference was found between the age of the patients and the perception of the usefulness of CPR, although a slightly younger age (46.3 ± 32.4) was observed in cases in which the doctors/nurses were sure that the resuscitation should not have been initiated. This aspect can be explained by the major problems presented by the patients, which clearly indicated that although they were younger, their conditions were very serious and CPR would not have the desired effect, as highlighted in Table 5 and Figure 1.

The last CA recalled was confirmed by witnesses in 42.59% of cases. In 35.2% of cases, CPR was initiated before the arrival of the advanced resuscitation team. A shockable heart rhythm was identified by the team only in 22.2% of cases, with a higher significant value (χ^2^ = 11.0348, *p* = 0.0261) of cases with shockable rhythm at which nurses were present (25.9%) compared to those found by doctors (18.52%). The main cause of CA was identified as cardiac in 57.41% of cases. The time interval of resuscitation lasted for an average of 37.07 ± 21.25 min, having a minimum value of 5 min and a maximum of 120 min. For 75% of patients, resuscitation took more than 25 min. The return of spontaneous circulation (ROSC) was obtained for 20.37% of cases, the declared discharge survival being 12.96%. A significant difference (*p* = 0.6711) was not identified when obtaining ROSC in the case of doctors (22.2%) compared to that reported by assistants (18.52%). In the analyzed group, no cases were identified for which there were restrictions on therapy (will, advanced directive, a mention “not to resuscitate”). The impressions regarding the physical condition of the patient did not have significant differences (χ^2^ = 4.3279, *p* = 0.3634) between doctors and nurses. For 64.81% of patients, the impression was bad or deficient and only in 27.78% of cases was it reasonable or good. In 92.59% of cases, the respondents appreciated that the resuscitation presented a good technical quality. This opinion was shared both by doctors and nurses, without significant differences (χ^2^ = 3.1409, *p* = 0.5345). Only 3.5% of the respondents were not satisfied with the technical quality of resuscitation.

We identified a significant association (χ^2^ = 6.9136, *p* = 0.01405) between ROSC and the perception of the utility of initiating resuscitation. Thus, it was found that in situations in which medical staff were sure that it was necessary to initiate resuscitation, the return of spontaneous circulation was noted in 23.36% of cases, and in 25% of cases where there was a certain insecurity. These results are shown in Table 6.

A statistically significant direct connection between the usefulness of CPR initiation and the initial heart rhythm of the patient (χ^2^ = 2.937, *p* = 0.8166) or survival at discharge (χ^2^ = 4.7168, *p* = 0.3176) was not identified.

We also performed a multivariate analysis regarding the perception of the utility of CPR and the patient-dependent variables (age, heart rate, ROSC and survival) in order to identify the presence of factors that influenced the perceptions of the medical personnel who are to initiate CPR. The results indicate that only the heart rhythm of the patient significantly influenced the perception of medical personnel with regard to the initiation of resuscitation (OR = 2.032, 95%CI: 1.866–4.771, *p* = 0.013). The constant significant parameter (*p* = 0.015) indicates that there may be other variables that have not been included in the study and which have a greater potential to change the perception of medical staff. We can appreciate the fact that a non-shockable heart rhythm can increase by 2.03 times the chances of the medical staff considering the initiation of CPR useless or being uncertain about the opportunity to initiate it. These results are shown in Table 7.

The opinion of the respondents (70.37%) was that they are often involved in CPR trials in which the efforts made are disproportionate to the expected prognosis of the patient in terms of survival or quality of life (*p* = 0.02142). However, in this category of patients, most respondents (87.04%) paid less attention to the quality of CPR maneuvers (*p* = 0.01248). In fact, 66.66% said that they most often do not have any doubt related to CPR in this category of patients, although 66.67% felt a state of emotional suffering, and 40.74% a state of moral suffering in such a situation. The pressure of situations of this nature cause the respondents to often feel the need to discuss the utility with co-workers (74.07%), and less frequently with people outside the service (25.93%).

## 4. Discussion

To the best of our knowledge, this is the first study conducted in the emergency department in Romania focused on the personnel involved in the care of patients during cardiac arrest, regarding the perception of the uselessness of CPR, exploring organizational, clinical and potentially relevant work factors, as well as specific factors related to patients. EMS staff were specifically asked about the latest cardiac arrest in which they were involved. As such, the reported prevalence of the perception of inadequate/unnecessary CPR was based on a specific clinical event attended by doctors and medical assistants, and not just a subjective general impression.

CA patients are particularly vulnerable to situations that put them at risk of dehumanization, as they do not have many of the specific characteristics of the human being: consciousness, purpose and determination. Moreover, those who have important cognitive, psychological and physical deficits, in the context of congenital or chronic conditions, may never recover them, thus prolonging their vulnerability to a lack of respect, often with profoundly negative effects for their relatives [21].

In this study, 54 respondents were analyzed, of which 50% were doctors and 50% were nurses, compared to the proportions of 36.9% and 25.1%, respectively, reported in the European REAPPROPRIATE study [22]. Of the respondents, 66.67% said they have a religious conviction; no elements have been found to suggest that this influences current medical practice. Regarding tension related to the work environment, we noticed that 59.25% of doctors believed that they are not appreciated by the public (*p* = 0.0291) compared to 74.07% of nurses. In addition, these categories of personnel consider their work in a resuscitation team to be very difficult (*p* = 0.0238). Moreover, 85.18% feel that their involvement and value in the resuscitation team are not recognized (*p* = 0.0118) and that they cannot express their opinions (88.9%) during the resuscitation, in relation to the opportunity of initiating CPR. However, only 5.56% said they want to leave their current profession, which can be explained by their attachment to the profession and the team, despite the dissatisfaction during the moments of the most serious emergency.

We found that 79.63% of the clinicians working in the Emergency Department of the County Clinical Hospital “St. Spiridon” who responded to the questionnaire perceived the last attempt by CPR as adequate. Moreover, we found that the presence of objective indicators of a weak prognosis did not change the prevalence of the perception of inadequate CPR. These percentages are similar to those from European studies that also included the data from Romania [22], in which 78.4% of the respondents considered the CPR to be adequate, 13.6% doubted the resuscitation indication and only 8% perceived it as inadequate/useless. Of those who felt that CPR should not have been initiated, doctors (22.22%) held a higher weight than nurses (3.70%) (*p* = 0.0103), compared to the European study, which did not identify a significant association between clinician profession and final perception [22].

We must mention that there is no concept of DNAR or advanced directives in the Romanian legislation, so all the results obtained in this study must be viewed from this perspective. This might explain why the clinicians did not consider important variables such as patient age, prior dementia, advanced stage cancer or quality of life before cardiopulmonary arrest, influencing the perception on the adequacy of CPR attempts. The results indicate that there is no significant difference between the age of the patients and the perception of the uselessness of CPR, although a slightly younger age was observed (46.3 ± 32.4) in instances when the doctors/nurses were sure that resuscitation should not have been initiated. This aspect can probably be explained by the severity of the problems presented by the patients, which clearly indicate that although the patients are younger, their conditions are very serious, and CPR will not provide the desired result and will not improve the quality of life. In patients with cardiac arrest with a non-shockable rhythm and without a witness, a group of patients known for their extremely unfavorable prognosis [11,23] and a low survival rate at discharge, regarding the early recognition of unnecessary CPR, 22.2% of respondents considered it very important and 46.59% important.

The results of this study indicate that only the heart rhythm of the patient significantly influenced the perception of the medical personnel in terms of the initiation of resuscitation (OR = 2.032, 95%CI: 1.866–4.771, *p* = 0.013). The constant significant parameter (*p* = 0.015) indicates that there may be other variables that have not been included in the study and which have a greater potential to change the perception of the medical staff. We can appreciate the fact that a non-shockable heart rhythm can increase by 2.03 times the chances of medical staff considering the initiation of CPR unnecessary or being uncertain about the opportunity to initiate it.

Unexpectedly, neoplasia in the terminal phase was considered an important situation in terms of inadequate CPR by only 64.82% of ED staff, without representing a factor that influences the perception of the utility of CPR. These findings indicate that most doctors and nurses either are not aware of the inadequacy of their CPR attempt or become aware of the potentially beneficial consequences of their actions in critical patient situations.

The other studied factors—the general quality of the CPR initiated by witnesses (75.93%), the initial non-shockable rhythm (64.81%), the presence of a DNAR order (85.18%) and comorbidities such as cancer (64.82%)—although they have been considered to be important in a larger proportion, did not present a statistical significance that would attest to their influence on the perception of CPR as useless. This may indicate that the perception of inadequacy is reduced and does not have the same meaning as in the case of professionals in Western European countries. It is important to evaluate the team leaders with regard to the uselessness of resuscitation, and to consider the opinion of all experienced members of the resuscitation team, regardless of their role.

The main limitation of this study based on a questionnaire is that the data volume is small, limited to only one ED in one area, but significant if we consider that this university ED is one of the largest in Romania. While the authors do not believe the structure of this population significantly differs from other large ED in terms of culture surrounding CPR practices, as local approaches to end-of-life care can vary, so can clinicians understand how DNAR orders are to be implemented [24].

A multidisciplinary care team’s questionnaire could give a more realistic picture on the perception of CPR futility. Given the significant knowledge gaps identified, a lack of targeted education may exacerbate misconceptions among medical providers about DNAR orders. Incorporation of palliative care education for all medical providers has been suggested by other authors included in the routine component of medical training [25].

While most of the previous studies focused on the perception of the practice of resuscitation performed by medical staff, the current study was the first to investigate the perception of inadequate/useless CPR, and the uncertainty in the initiation of CPR based on the recall of the last resuscitation performed by clinicians, doctors and nurses.

## 5. Conclusions

The objective criteria related to the patient were the most important predictors for appreciating the correctness of the decision to initiate CPR, independent of the clinician’s profession. Despite the concordances observed between the clinician’s perception and the result of the patient’s resuscitation, the analysis of unfavorable prognostic indicators revealed that the presence of a non-shockable rhythm is the only variable that can increase by two times the chances of medical staff considering the initiation of CPR unnecessary or being unsure about the opportunity to initiate it.

Although we identified a high level of dissatisfaction regarding work as part of a resuscitation team (85.15% of respondents believed their work was hard and 85.18% of respondents did not feel appreciated) and that more than half of the respondents said they often wonder how adequate a resuscitation attempt is, most of the respondents (79.63%) considered it necessary to start resuscitation, and only 3.5% of the respondents were not satisfied with the technical quality of the resuscitation.

The results of this study demonstrate a tendency toward the constraining rules and conservative practices of the personnel that intervene in cardiopulmonary resuscitation. This can have negative consequences for patients, who give up their right to die with dignity and cannot express their end-of-life desire, on the family and on society, which is not prepared to compensate for the effects of the unnecessary resuscitation of patients with a very low quality of life. From this perspective, it is important to implement the notions of DNAR orders or advanced directives in Romanian legislation.

For the medical personnel who are part of resuscitation teams, extended in-depth studies are required to confirm if the elements of unfavorable prognosis influence the attitude of the team, and to establish correlations with ROSC rate, survival and the evolution of patients with cardiorespiratory arrest.

## Figures and Tables

**Table 1:** Workplace tensions.

	Totally Disagree n (%)	Disagree n (%)	Agree n (%)	Totally Agree n (%)	Pearson Chi-Square Test Spearman Correlation
I have to work hard					
Nurses	1 (3.70)	5 (18.52)	15 (55.56)	6 (22.22)	χ^2^ = 9.4530, *p* = 0.02384 r = 0.4347
Doctors	2 (7.41)	0 (0.00)	13 (48.15)	12 (44.44)	
Total	3 (5.56)	5 (9.26)	28 (51.85)	18 (33.33)	
I am required to do an excessive amount of work					
Nurses	3 (11.11)	8 (29.63)	11 (40.74)	5 (18.52)	χ^2^ = 5.9169, *p* = 0.1157 r = 0.2479
Doctors	0 (0.00)	5 (18.52)	14 (51.85)	8 (29.63)	
Total	3 (5.56)	13 (24.07)	25 (46.3)	13 (24.07)	
I do not have enough time to finish my work					
Nurses	5 (18.52)	11 (40.74)	11 (40.74)	0 (0.00)	χ^2^ = 12.21, *p* = 0.00226 r = 0.7322
Doctors	1 (3.70)	4 (14.81)	17 (62.96)	5 (18.52)	
Total	6 (11.11)	15 (27.78)	28 (51.85)	5 (9.26)	
I do not have to do repetitive work too often					
Nurses	0 (0.00)	14 (51.85)	12 (44.44)	1 (3.70)	χ^2^ = 10.0538, *p* = 0.01812 r = −0.3135
Doctors	5 (18.52)	13 (48.15)	6 (22.22)	3 (11.11)	
Total	5 (9.26)	27 (50.00)	18 (33.33)	4 (7.41)	
I have to learn new things					
Nurses	0 (0.00)	2 (7.41)	11 (40.74)	14 (51.85)	χ^2^ = 3.3801, *p* = 0.3665 r = 0.2088
Doctors	1 (3.70)	2 (7.41)	6 (22.22)	18 (66.67)	
Total	1 (1.85)	4 (7.41)	17 (31.48)	32 (59.26)	
I have great freedom in deciding how to do my job					
Nurses	3 (11.11)	14 (51.85)	7 (25.93)	3 (11.11)	χ^2^ = 10.37, *p* = 0.9913 r = 0.04439
Doctors	3 (11.11)	13 (48.15)	8 (29.63)	3 (11.11)	
Total	6 (11.11)	27 (50.00)	15 (27.78)	6 (11.11)	
I work with people who are willing to help when needed					
Nurses	0 (0.00)	0 (0.00)	12 (44.44)	15 (55.56)	χ^2^ = 9.2500, *p* = 0.02615 r = −0.5646
Doctors	1 (3.70)	4 (14.81)	14 (51.85)	8 (29.63)	
Total	1 (1.85)	4 (7.41)	26 (48.15)	23 (42.59)	
I am thinking of leaving my current job / current position					
Nurses	15 (55.56)	10 (37.04)	2 (7.41)	0 (0.00)	χ^2^ = 2.4728, *p* = 0.3936 r = 0.3408
Doctors	10 (37.04)	13 (48.15)	3 (11.11)	1 (3.70)	
Total	25 (46.03)	23 (42.59)	5 (9.26)	1 (1.85)	
I am thinking of leaving my current profession					
Nurses	19 (70.37)	8 (29.63)	0 (0.00)	0 (0.00)	χ^2^ = 5.7662, *p* = 0.03596 r = 0.4720
Doctors	13 (48.15)	11 (40.74)	3 (11.11)	0 (0.00)	
Total	32 (59.26)	19 (35.19)	3 (5.56)	0 (0.00)	

**Table 2:** Team performance during resuscitation.

	Totally Disagree n (%)	Disagree n (%)	Agree n (%)	Totally Agree n (%)	Pearson Chi-Square Test Spearman Correlation
I feel valuable and appreciated within the resuscitation team					
Nurses	3 (11.11)	21 (77.78)	3 (11.11)	0 (0.00)	χ^2^ = 9.273, *p* = 0.0118 r = 0.4818
Doctors	8 (29.63)	14 (51.85)	5 (18.52)	0 (0.00)	
Total	11 (20.37)	35 (64.81)	8 (14.81)	0 (0.00)	
I am confident in expressing my concerns about how appropriate a resuscitation is					
Nurses	5 (18.52)	19 (70.37)	3 (11.11)	0 (0.00)	χ^2^ = 6053, *p* = 0.73883 r = 0.1568
Doctors	3 (11.11)	21 (77.78)	3 (11.11)	0 (0.00)	
Total	8 (14.81)	40 (74.07)	6 (11.11)	0 (0.00)	
The team leader shows his appreciation for the efforts made regardless of the result					
Nurses	2 (7.41)	15 (55.56)	10 (37.04)	0 (0.00)	χ^2^ = 76674, *p* = 0.6815 r = −0.1401
Doctors	4 (14.81)	14 (51.85)	9 (33.33)	0 (0.00)	
Total	6 (11.11)	29 (53.70)	19 (35.19)	0 (0.00)	
During resuscitation, the presence of the family is ensured					
Nurses	9 (33.33)	15 (55.56)	1 (3.70)	2 (7.41)	χ^2^ = 8.776, *p* = 0.012 r = 0.5968
Doctors	8 (29.63)	14 (51.85)	5 (18.52)	0 (0.00)	
Total	17 (31.48)	29 (53.70)	6 (11.11)	2 (3.70)	
After a resuscitation, a special time is granted for debriefing					
Nurses	2 (7.41)	9 (33.33)	13 (48.15)	3 (11.11)	χ^2^ = 5.3675, *p* = 0.0146 r = 0.6627
Doctors	1 (3.70)	8 (29.63)	18 (66.67)	0 (0.00)	
Total	3 (5.56)	17 (31.48)	31 (57.41)	3 (5.56)	
I often wonder how appropriate a resuscitation attempt is					
Nurses	1 (3.70)	11 (40.74)	13 (48.15)	2 (7.41)	χ^2^ = 2.4389, *p* = 0.4693 r = 0.1764
Doctors	1 (3.70)	10 (37.04)	10 (37.04)	6 (22.22)	
Total	2 (3.70)	21 (38.89)	23 (42.59)	8 (14.81)	

**Table 3:** Factors influencing perceptions regarding the opportunity for resuscitation.

	Not Important	Less Important	Important	Very Important	Pearson Chi-Square Test Spearman Correlation
The general quality of the CPR before your arrival is weak					
Nurses	4 (14.81)	2 (7.41)	10 (37.04)	11 (40.74)	χ^2^ = 0.8705, *p* = 0.8325 r = −0.0541
Doctors	3 (11.11)	4 (14.81)	10 (37.04)	10 (37.04)	
Total	7 (12.96)	6 (11.11)	20 (37.04)	21 (38.89)	
The initial rhythm is non-shock					
Nurses	7 (25.93)	4 (14.81)	9 (33.33)	7 (25.93)	χ^2^ = 3.1875, *p* = 0.3718 r = 0.09677
Doctors	3 (11.11)	5 (18.52)	14 (51.85)	5 (18.52)	
Total	10 (18.52)	9 (16.67)	23 (42.59)	12 (22.22)	
The presence of an order stating “not to resuscitate” (DNAR—do not attempt resuscitation)					
Nurses	2 (7.41)	1 (3.70)	7 (25.93)	17 (62.96)	χ^2^ = 2.3914, *p* = 0.4853 r = −0.2104
Doctors	2 (7.41)	3 (11.11)	10 (37.04)	12 (44.44)	
Total	4 (7.41)	4 (7.41)	17 (31.48)	29 (53.70)	
The patient is very old					
Nurses	11 (40.74)	4 (14.81)	6 (22.22)	6 (22.22)	χ^2^ = 3.5768, *p* = 0.3109 r = −0.1181
Doctors	10 (37.04)	8 (29.63)	7 (25.93)	2 (7.41)	
Total	21 (38.89)	12 (22.22)	13 (24.07)	8 (14.81)	
The patient suffers from dementia					
Nurses	18 (66.67)	2 (7.41)	6 (22.22)	1 (3.70)	χ^2^ = 7.1827, *p* = 0.0242 r = 0.6287
Doctors	13 (48.15)	7 (25.93)	5 (18.52)	2 (7.41)	
Total	31 (57.41)	9 (16.67)	11 (20.37)	3 (5.56)	
Patient suffers from advanced stage cancer					
Nurses	7 (25.93)	3 (11.11)	9 (33.33)	8 (29.63)	χ^2^ = 1.6010, *p* = 0.6591 r = 0.04708
Doctors	4 (14.81)	5 (18.52)	11 (40.74)	7 (25.93)	
Total	11 (20.37)	8 (14.81)	20 (37.04)	15 (27.78)	
Patient has poor quality of life before installing cardiorespiratory arrest					
Nurses	20 (74.07)	1 (3.70)	3 (11.11)	3 (11.11)	χ^2^ = 18.1748, *p* = 0.00029 r = 0.5639
Doctors	6 (22.22)	10 (37.04)	8 (29.63)	3 (11.11)	
Total	26 (48.15)	11 (20.37)	11 (20.37)	6 (11.11)	

**Table 4:** Factors influencing the perception of the adequacy of a resuscitation.

	Totally Disagree	Disagree	Agree	Totally Agree	Pearson Chi-Square Test Spearman Correlation
The initial rhythm is shockable					
Nurses	5 (18.52)	0 (0.00)	8 (29.63)	14 (51.85)	χ^2^ = 7.7569, *p* = 0.01229 r = 0.1312
Doctors	1 (3.70)	2 (7.41)	9 (33.33)	15 (55.56)	
Total	6 (11.11)	2 (3.70)	17 (31.48)	29 (53.70)	
The patient is a possible organ donor					
Nurses	0 (0.00)	1 (3.70)	19 (70.37)	7 (25.93)	*χ^2^* = 6.7382, *p* = 0.0154 r = 0.2542
Doctors	0 (0.00)	5 (18.52)	14 (51.85)	8 (29.63)	
Total	0 (0.00)	6 (11.11)	33 (61.11)	15 (27.78)	
The patient is pregnant					
Nurses	0 (0.00)	0 (0.00)	5 (18.52)	22 (81.48)	χ^2^ = 4.32779, *p* = 0.0194 r = −0.3151
Doctors	2 (7.41)	0 (0.00)	3 (11.11)	22 (81.48)	
Total	2 (3.70)	0 (0.00)	8 (14.81)	44 (81.48)	
The patient is a child					
Nurses	0 (0.00)	0 (0.00)	1 (3.70)	26 (96.30)	χ^2^ = 4.647, *p* = 0.0161 r = −0.7124
Doctors	1 (3.70)	0 (0.00)	4 (14.81)	22 (81.48)	
Total	1 (1.85)	0 (0.00)	5 (9.26)	48 (88.89)	

**Table 5:** Statistical indicators of patients’ age according to the perception of the utility of CPR.

Medical Staff Opinion	Mean Age	Mean		SD	Min	Max	Q25	Median	Q75
		−95%	−95%						
O1	53.8	46.1	61.6	25.2	1.0	87.0	45.0	62.0	70.0
O2	65.3	48.4	82.1	10.6	54.0	77.0	56.5	65.0	74.0
O3	46.3	16.3	76.3	32.4	1.0	75.0	1.0	65.0	70.0

Levene Test of Homogeneity of Variances: F = 1.8376, *p* = 0.1695 (*). Marked effects are significant at *p* < 0.05. Legend: O1. I have totally agreed to start resuscitation. O2. I was uncertain if the resuscitation had to be initiated. O3. I was sure that resuscitation should not have been initiated.

**Table 6:** The perception of the utility of the initiation of CPR vs. ROSC.

Opinion on Necessity of CPR	I Do Not Know	No	Yes	Total
I totally agreed to start resuscitation	6	27	10	43
I was unsure that CPR should have been initiated	0	3	1	4
I was sure that CPR should not have been initiated	0	7	0	7

Pearson Chi-square test: X = 6.9136, *p* = 0.014053 *, Spearman correlation: r = 0.3751, (*) marked effects are significant at *p* < 0.05.

**Table 7:** Logistic regression regarding the perception of the need to start resuscitation vs. variables dependent on the patient.

Test for the Validity of the Model (“Enter” Method), Hosmer–Lemeshow							
Step	Chi-square (test, χ^2^)		Df (degrees of freedom)			Sig.p (significance level)	
1	1.381		4			0.2547	
Results verifying the prediction power of the model, model summary							
Step	−2 Log likelihood		Cox and Snell R Square			Nagelkerke R Square	
1	0.2427		0.7275			0.8512	
**Multivariate analysis** **The perception of the** **necessity of CPR vs.**	**Beta**	**SE**	**Wald**	**Sig.p**	**Odd** **Ratio** **Exp(β)**	**95% CI for Exp(B)**	
						**Lower**	**Upper**
Age	0.007	0.012	0.394	0.530	1.007	0.985	1.031
Cardiac rhythm	1.709	0.035	2.654	0.013 *	2.032	1.866	4.771
ROSC	−0.532	0.046	0.951	0.330	0.587	0.202	1.712
Constant	−1.799	1.273	1.998	0.015 *	2.165		

χ^2^ = 0.853 (the fit degree of the model); *df* = 3; *p =* 0.837; 95% CI. CI—confidence interval, df—degrees of freedom, OR—odds ratio, SE—standard error. * marked effects are significant at *p* < 0.05.

## Data Availability

Not applicable.
